# Sublobar Resection in Stage I Lung Cancer With Tumor Spread Through Air Spaces

**DOI:** 10.1016/j.atssr.2025.01.010

**Published:** 2025-02-13

**Authors:** Andrea L. Axtell, Brittany Walker, Joshua R. Brady, Jocelyn C. Zajac, Daniel P. McCarthy, James D. Maloney, Malcolm M. DeCamp

**Affiliations:** 1 Division of Cardiothoracic Surgery, Department of Surgery, University of Wisconsin School of Medicine and Public Health, Madison, Wisconsin

## Abstract

**Background:**

Stage I lung cancer is increasingly being treated with sublobar resection. However, it is unknown whether patients with airspace invasion derive similar benefits. We therefore analyzed the association between tumor spread through air spaces (STAS) and survival.

**Methods:**

A retrospective cohort analysis was conducted on 421 patients who underwent a lung cancer resection between 2018 and 2022 at an academic institution. Baseline characteristics were compared between patients who did and did not have STAS. Overall survival and disease recurrence were analyzed using Kaplan-Meier and Cox models.

**Results:**

Of 421 patients who underwent lung cancer resection, 97 (23%) had STAS. There was no difference in STAS based on comorbidities or pulmonary function, however, patients with STAS were more likely to have higher pack-year smoking histories (47 vs 40 years, *P* = .041). Patients with STAS were more likely to have adenocarcinoma (91% vs 78%, *P* = .049), larger tumor size (2.6 vs 2.2 cm, *P* = .016), and lymphovascular invasion (46% vs 32%, *P* = .012). In patients with stage I disease, those with STAS who underwent sublobar resection had decreased overall survival compared with those without STAS (*P* = .042) or those who underwent lobectomy, regardless of the presence or absence of STAS. Five-year overall survival was 73% for stage I patients with STAS who underwent sublobar resection compared with 87% in patients without STAS, and 90% in patients without STAS who underwent lobectomy.

**Conclusions:**

In patients with stage I disease, STAS is associated with decreased overall survival in patients who undergo sublobar resection; however, STAS does not affect overall survival after lobectomy.

## Visual Abstract

**Figure:**
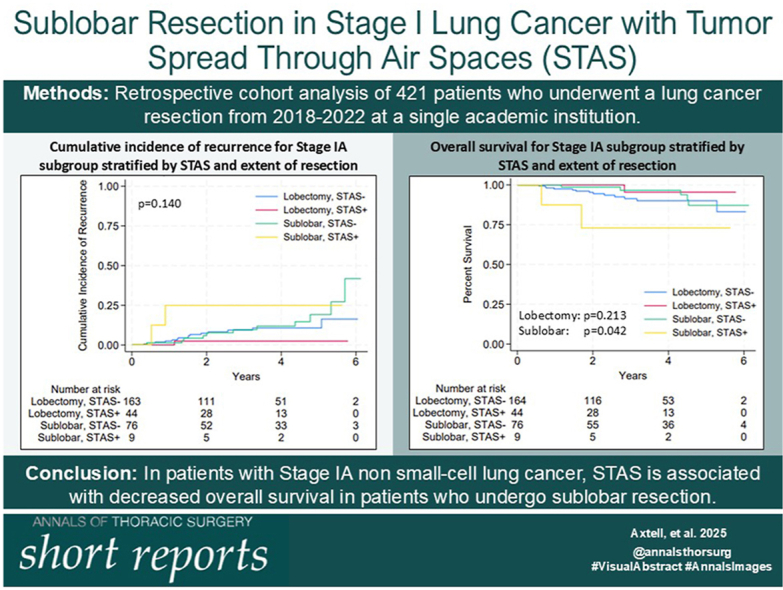

**In Short**

▪ Tumor spread through air spaces is associated with pulmonary adenocarcinoma with papillary and micropapillary features.
▪ In patients with stage I disease, tumor spread through air spaces is associated with decreased overall survival in patients who undergo sublobar resection compared with lobectomy.

Airspace invasion—defined as spread of micropapillary clusters, solid nests, or single cancer cells into air spaces in the lung parenchyma beyond the edge of the main tumor—is an important mechanism of invasion with significant prognostic and clinical implications.^1^ Spread through air spaces (STAS) has been predominantly described in lung adenocarcinoma and in a review of resected stage I lung adenocarcinomas^2^; the presence of STAS was demonstrated in up to 38% of cases. Two recent international randomized controlled trials have compared pulmonary lobectomy to sublobar resection (wedge vs segmentectomy) for peripheral stage IA non-small cell lung cancer (NSCLC).^3^^,^^4^ Disease-free survival at 5 years was not significantly different between patients who underwent lobectomy compared to segmentectomy in 1 trial, and, in the other, 5-year overall survival was higher in the segmentectomy group compared with the lobectomy group. These studies support the utilization of parenchymal-sparing sublobar resection to treat stage IA NSCLC. However, histopathologic subtypes were not reported in either study, and it remains to be proven whether patients with STAS derive similar benefits.

We therefore aimed to analyze a cohort of patients with surgically resected NSCLC to determine the association between tumor STAS and disease recurrence and overall survival, specifically in patients with stage IA disease who underwent sublobar vs lobar resection.

## Patients and Methods

A retrospective cohort analysis using an institutional database was conducted on adult patients who underwent a first-time pulmonary resection for NSCLC between January 2018 and June 2022. All malignant NSCLC histologies and stages IA-IIIA disease were included. Disease stage was based on the 8th edition of the American Joint Committee on Cancer TNM Staging Manual. Patients who underwent emergency resections and those with missing data for the presence or absence of STAS were excluded.

Baseline clinical, operative, and pathologic characteristics were compared between patients with and without STAS using a Student’s *t* test or Mann-Whitney U test for continuous variables and a χ^2^ test or Fisher exact test for categorical variables. Overall survival for patients with and without STAS was determined using the Kaplan-Meier method followed by a log-rank test for differences between groups. To address variables that confound the relationships between STAS and overall survival, a multivariable Cox proportional hazards regression model was developed to identify independent predictors of mortality. Cumulative incidence of recurrence was analyzed using a competing risks method accounting for death without recurrence as a competing event.

A predefined subgroup analysis was also conducted in patients with stage IA disease with tumors ≤2.0 cm. Overall survival and disease recurrence were compared between patients with and without STAS who underwent lobectomy vs a sublobar resection (defined as either a wedge resection or a segmentectomy). All analyses were completed using STATA v17.0 (STATA Corp). A *P* value of less than .05 was considered statistically significant.

## Results

Of 421 patients who underwent a pulmonary resection for stage IA-IIIA NSCLC, 97 (23%) had STAS on final pathologic assessment. Baseline demographic and clinical characteristics for patients with and without STAS are presented in Table 1. There was no difference in STAS based on age, sex, or patient comorbidities. Histologically, patients with STAS were more likely to have adenocarcinoma with micropapillary and papillary predominant features (Supplemental Figure 1) and a larger mean tumor size (2.6 cm vs 2.2 cm, *P* = .016) (Table 2). Tumors with STAS had more concurrent lymphovascular invasion (32% vs 46%, *P* = .012) and a more advanced pathologic stage (*P* = .003).

**Table 1:** Baseline Clinical Characteristics

Characteristic	Entire Cohort			Stage IA Subgroup		
	No STAS (n = 324)	STAS + (n = 97)	*P* Value	No STAS (n = 240)	STAS + (n = 53)	*P* Value
Age, y	68 ± 9	67 ± 10	.206	68 ± 8	66 ± 7	.249
Female sex	188 (58)	57 (59)	.897	148 (62)	34 (64)	.762
BMI >30	91 (28)	36 (37)	.089	63 (26)	16 (30)	.570
Smoking history			.198			.487
Never	54 (17)	22 (22)		41 (17)	10 (19)	
Current	92 (28)	20 (21)		69 (29)	11 (21)	
Former	178 (55)	55 (57)		130 (54)	32 (60)	
Pack year history	40 ± 25	47 ± 32	.041	40 ± 24	46 ± 37	.166
Hypertension	215 (66)	67 (69)	.618	160 (67)	39 (74)	.348
Coronary artery disease	65 (20)	18 (19)	.744	46 (19)	11 (21)	.802
Congestive heart failure	5 (2)	3 (3)	.393	4 (2)	2 (4)	.299
Diabetes mellitus	52 (16)	19 (20)	.414	35 (15)	8 (15)	.933
Pulmonary hypertension	15 (5)	3 (3)	.775	12 (5)	2 (4)	1.000
COPD	116 (36)	39 (40)	.430	88 (37)	21 (40)	.703
Interstitial fibrosis	6 (2)	3 (3)	.436	5 (2)	1 (2)	1.000
Pulmonary function tests						
FEV_1_, predicted %	88 ± 19	90 ± 18	.242	88 ± 20	91 ± 17	.380
DLCO, predicted %	76 ± 20	83 ± 18	.004	76 ± 20	84 ± 19	.034

Values are presented as n (%) or mean ± standard deviation.

BMI, body mass index; COPD, chronic obstructive pulmonary disease; DLCO, diffusing capacity of lung for carbon monoxide; FEV_1_, forced expiratory volume in 1 second; STAS, spread through air spaces.

**Table 2:** Clinicopathologic Features and Operative Characteristics

Characteristic	Entire Cohort			Stage IA Subgroup		
	No STAS (n = 324)	STAS + (n = 97)	*P* Value	No STAS (n = 240)	STAS + (n = 53)	*P* Value
Histology			.049			.682
Adenocarcinoma	254 (78)	88 (91)		196 (82)	48 (91)	
Squamous cell carcinoma	63 (19)	9 (9)		39 (16)	5 (9)	
Large cell carcinoma	3 (0)	0 (0)		2 (1)	0 (0)	
Histologic subtype			.013			.019
Lepidic	33 (13)	4 (5)		32 (16)	2 (4)	
Acinar	155 (61)	50 (57)		119 (61)	26 (55)	
Papillary	7 (3)	8 (9)		6 (3)	6 (13)	
Solid	27 (11)	10 (11)		17 (9)	7 (15)	
Micropapillary	3 (1)	4 (5)		3 (1)	1 (2)	
Mucinous	28 (11)	11 (13)		19 (10)	5 (11)	
Tumor size, cm	2.2 ± 1.4	2.6 ± 1.9	.016	1.8 ± 0.8	1.8 ±0.7	.628
Margin distance, cm	2.7 ± 1.9	2.7 ± 1.7	.832	2.9 ± 1.9	2.9 ± 1.8	.816
Visceral pleural invasion	99 (31)	34 (35)	.403	59 (25)	12 (23)	.754
Lymphovascular invasion	101 (32)	45 (46)	.012	58 (25)	15 (28)	.619
Pathologic stage			.003	n/a	n/a	
Stage I	239 (76)	53 (58)				
Stage IIA	10 (3)	2 (2)				
Stage IIB	62 (20)	35 (39)				
Stage IIIA	5 (1)	1 (1)				
PD-L1 status			.196			.630
<1%	51 (16)	21 (22)		39 (16)	11 (21)	
1-50%	78 (24)	28 (29)		47 (20)	9 (17)	
>50%	25 (8)	9 (9)		14 (6)	5 (9)	
Unknown	170 (52)	39 (40)		139 (58)	28 (53)	
Neoadjuvant therapy	12 (4)	3 (3)	1.000	n/a	n/a	
Operation			.270			.082
Wedge resection	23 (7)	6 (6)		17 (7)	1 (2)	
Segmentectomy	62 (19)	12 (12)		59 (25)	8 (15)	
Lobectomy	239 (74)	79 (81)		163 (88)	44 (83)	

Values are presented as n (%) or mean ± standard deviation.

PD-L1, Programmed Death Ligand 1; STAS, spread through air spaces.

**Figure 1:**
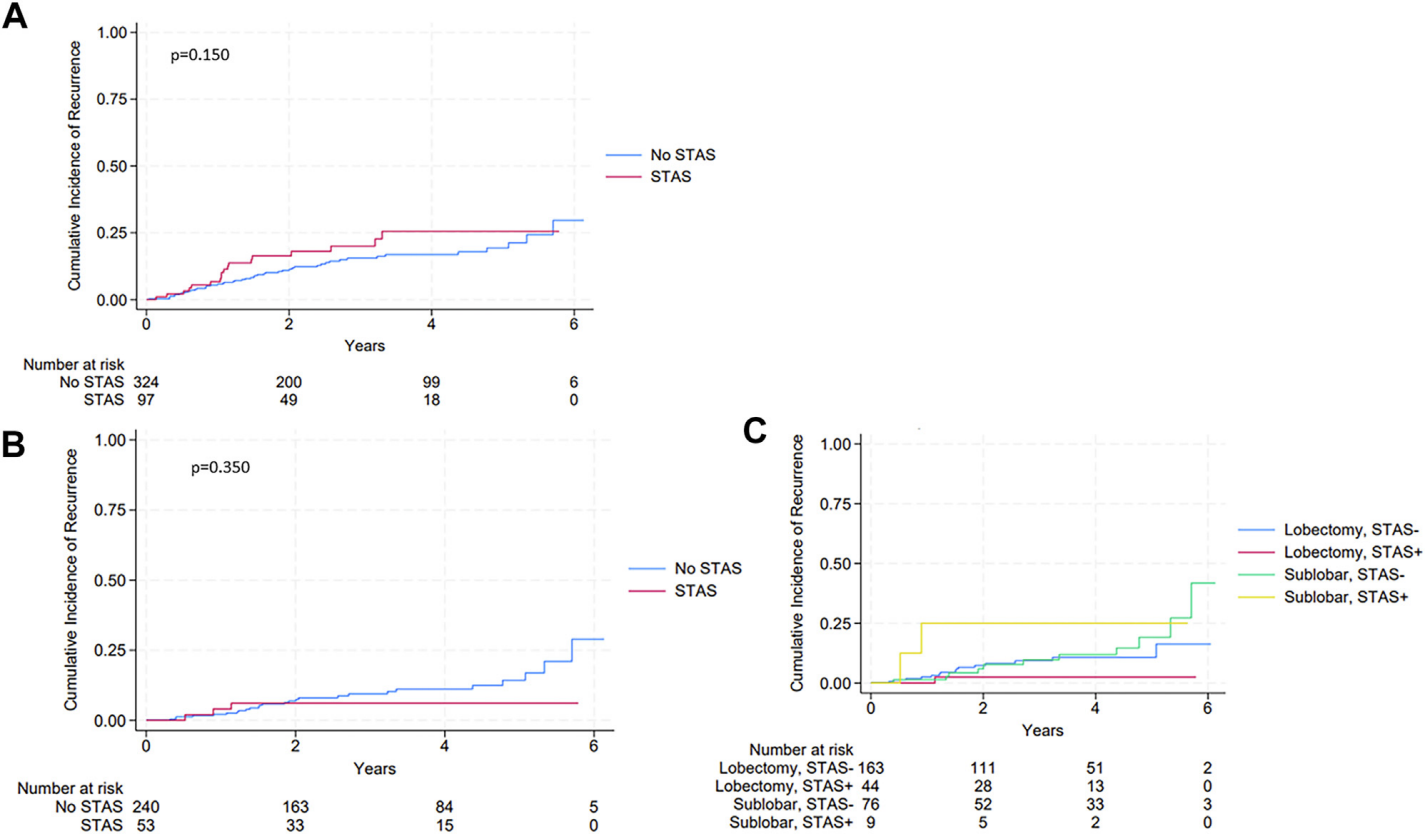
Cumulative incidence of recurrence. (A) Entire cohort. (B) Stage 1 disease only. (C) Stage 1 disease, stratified by tumor spread through air spaces (STAS) and the extent of resection.

There was no difference in the location of recurrence (Supplemental Table 1) or the cumulative incidence of recurrence (*P* = .15) (Figure 1A) in the entire cohort of STAS-negative vs STAS-positive tumors. In the subgroup of patients with stage IA disease and tumors <2 cm (Figures 1B, 1C), there was a trend toward an increased incidence of recurrence in patients with STAS-positive tumors who underwent sublobar resection (n = 9) compared with lobectomy (n = 44), although this did not reach statistical significance and was not associated with margin distance (Supplemental Figure 2). In patients with STAS-negative tumors, there was no difference in the cumulative incidence of recurrence based on whether the patient underwent sublobar vs lobar resection.

**Figure 2:**
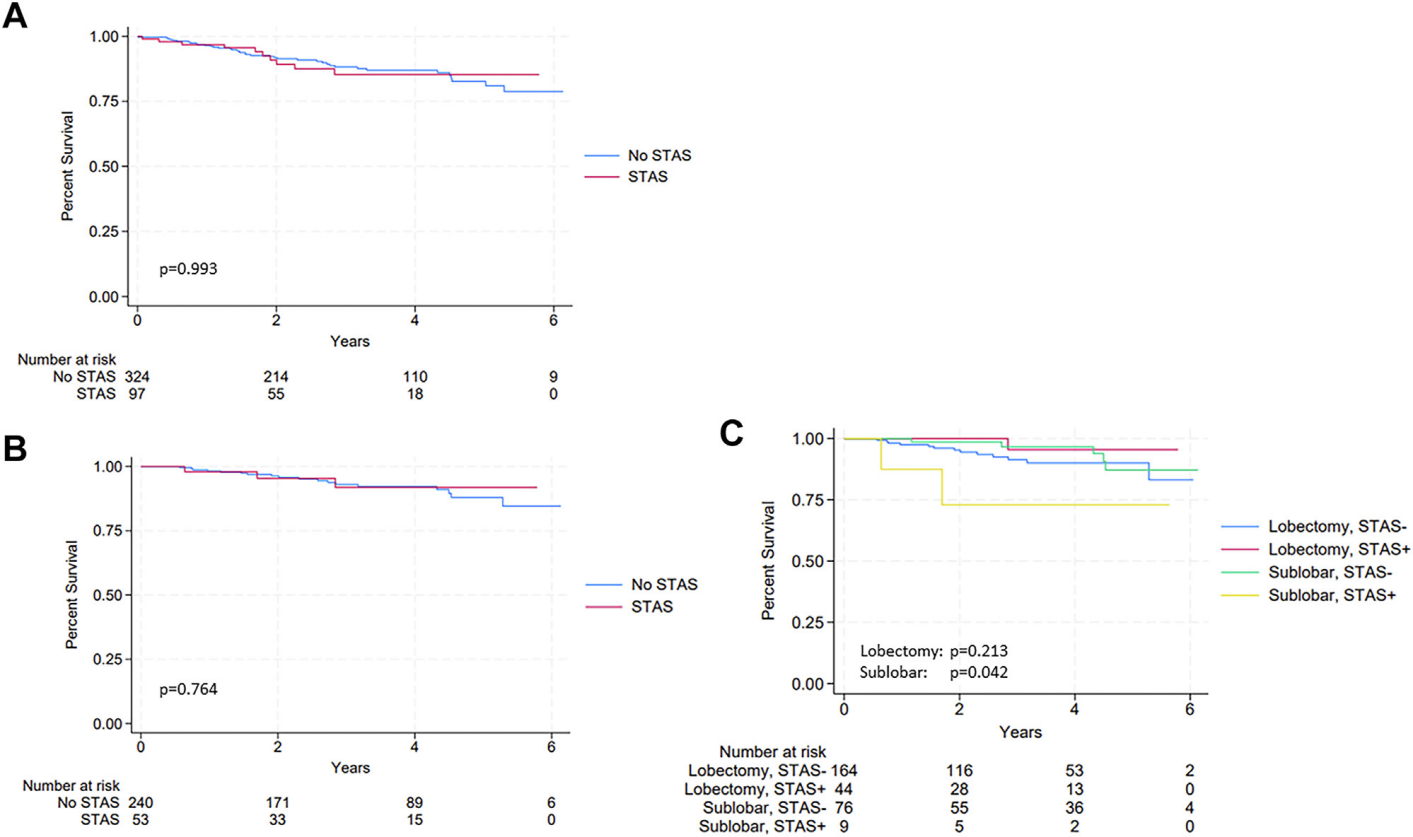
Overall survival. (A) Entire cohort. (B) Stage 1 disease only. (C) Stage 1 disease, stratified by tumor spread through air spaces (STAS) and the extent of resection.

There was no difference in 5-year or overall survival in the entire cohort of STAS-negative versus STAS-positive tumors (*P* = .993) (Figure 2A). In the subgroup of patients with stage IA disease and tumors <2 cm (Figures 2B, 2C), there was significantly decreased overall survival in patients with STAS-positive tumors who underwent sublobar resection compared with patients with STAS-positive tumors who underwent lobectomy (*P* = .042). For patients with stage IA disease with tumors <2 cm that were STAS-negative, there was no difference in overall survival for those who underwent sublobar compared with lobar resection (*P* = .213.) Five-year overall survival was 73% for stage IA patients with STAS who underwent sublobar resection compared with 95% in those with STAS who underwent lobectomy. On multivariable analysis (Supplemental Table 2), advanced pathologic stage (hazard ratio for IIA compared with I, 5.7 [95% CI, 1.2-27.5], *P* = .028) was a predictor of mortality, whereas Programmed Death Ligand 1 expression >1% was protective (hazard ratio, 0.4 [95% Ci, 0.2-0.9], *P* = .048).

## Comment

In this analysis of surgically resected stage IA-IIIA NSCLC, we identify a 23% occurrence of tumor STAS. We demonstrate that STAS is not associated with patient age or underlying pulmonary parenchymal comorbidities; however, it is associated with adenocarcinoma with more aggressive histologic subtypes including micropapillary and papillary predominant features, as well as larger tumors (80% of T4 tumors were STAS-positive compared with 20% of T1 tumors). Previous work^5^ has also identified solid tumors and those with maximum standardized uptake value >2.5 to be associated with STAS. In the subgroup of patients with stage IA disease and tumors <2 cm, the presence of STAS increased the cumulative incidence of recurrence and decreased overall survival in patients who underwent sublobar resection compared with lobectomy. In those patients who underwent lobectomy, there was no difference in disease recurrence or overall survival based on the presence or absence of STAS at any stage of disease.

This study is consistent with prior analyses identifying STAS as an important prognostic factor for overall survival. For example, Warth and colleagues^6^ reported a reduction in mean overall survival from 71 months in patients without STAS to 54 months in patients with STAS and found this trend to be consistent across all stages of disease. Additionally, a meta-analysis^7^ of 10 studies including 2974 patients demonstrated a pooled hazard ratio for mortality of 1.75 (95% CI, 1.37-2.22, *P* < .001) for patients with STAS-positive tumors compared with patients without STAS. In an analysis of the International Association for the Study of Lung Cancer database, STAS was associated with significantly worse recurrence-free and overall survival for all NSCLC histologic types and extent of resection (lobar and sublobar).^8^

Although our data suggest an increased incidence of recurrence in patients with STAS who underwent a sublobar resection, this did not reach statistical significance given a small sample size. This is in contrast to previous analyses,^9^^,^^10^ which have reported a significantly increased risk of locoregional recurrence in patients with early-stage disease and STAS-positive tumors. In an analysis by Kadota and colleagues,^9^ there was a 5-year recurrence-free probability of 57% in patients with STAS-positive tumors who underwent sublobar resection compared with 77% in those who underwent lobectomy (*P* = .002); however, there was no difference in the risk of distant recurrence based on the extent of resection. In an analysis by Khalil and colleagues,^10^ STAS-positive tumors were associated with a doubling of locoregional and distant recurrences over 10 years; lobar resection did not confer a survival advantage in this study, however.

This study should be interpreted in the context of several limitations. First, it represents a single academic institution’s experience. Findings, therefore, may have limited generalizability to the larger population. Second, systematic institutional reporting of STAS did not begin until 2018, limiting the sample size available for analysis. Third, the cumulative incidence of recurrence may be underrepresented as only recurrences identified in or reported to our health system were captured. Finally, the time period included predates the publication of the JCOG (Japan Clinical Oncology Group)^4^ and CALGB (Cancer and Leukemia Group B) trials^3^ supporting sublobar resection, which may confound the overall survival of patients selected for sublobar resection.

In conclusion, tumor STAS is associated with pulmonary adenocarcinoma with papillary and micropapillary features. Based on this analysis and previous literature, STAS is associated with an increased incidence of recurrence and may decrease overall survival in early-stage patients who undergo sublobar resection compared with lobectomy.

## References

[1] Shih A.R., Mino-Kenudson M. (2020). Updates on spread through air spaces (STAS) in lung cancer. Histopathology.

[2] Kadota K., Nitadori J.I., Sima C.S. (2015). Tumor spread through air spaces is an important pattern of invasion and impacts the frequency and location of recurrences after limited resection for small stage I lung adenocarcinomas. J Thorac Oncol.

[3] Altorki N., Wang X., Kozono D. (2023). Lobar or sublobar resection for peripheral stage IA non-small-cell lung cancer. N Engl J Med.

[4] Saji H., Okada M., Tsuboi M. (2022). Segmentectomy versus lobectomy in small-sized peripheral non-small-cell lung cancer (JCOG0802/WJOG4607L). Lancet.

[5] Tasnim S., Raja S., Mukhopadhyay S. (2024). Preoperative predictors of spread through air spaces in lung cancer. JTCVS.

[6] Warth A., Muley T., Kossakowski C.A. (2015). Prognostic impact of intra-alveolar tumor spread in pulmonary adenocarcinoma. Am J Surg Pathol.

[7] Chen D., Mao Y., Wen J. (2019). Tumor spread through air spaces in non-small cell lung cancer: a systematic review and meta-analysis. Ann Thorac Surg.

[8] Travis W.D., Eisele M., Nishimura K.K. (2024). The IASLC staging project for lung cancer. J Thorac Oncol.

[9] Kadota K., Kushida Y., Kagawa S. (2019). Limited resection is associated with a higher risk of locoregional recurrence than lobectomy in stage I lung adenocarcinoma with tumor spread through air spaces. Am J Surg Pathol.

[10] Khalil H.A., Shi W., Mazzola E. (2023). Analysis of recurrence in lung adenocarcinoma with spread through air spaces. J Thorac Cardiovasc Surg.

