# Supplementary material for: Sublobar Resection in Stage I Lung Cancer With Tumor Spread Through Air Spaces
Source: Ann Thorac Surg Short Rep. 2025 Feb 13;3(3):687–92. doi: 10.1016/j.atssr.2025.01.010 (PMC12559274; doi:10.1016/j.atssr.2025.01.010)
Supplement: Supplementary Material [file mmc1.docx]

**Supplemental Material**

**Supplemental Tables**

**Table S1: Recurrence Patterns**

|  | **All Stages** | | | **Stage 1** | | |
| --- | --- | --- | --- | --- | --- | --- |
|  | **No STAS (n=324)** | **STAS + (n=97)** | **p-value** | **No STAS (n=240)** | **STAS + (n=53)** | **p-value** |
| Recurrence | 48 (15%) | 18 (19%) | 0.374 | 26 (11%) | 3 (6%) | 0.254 |
| Local | 22 (7%) | 4 (4%) |  | 15 (6%) | 0 (0%) |  |
| Regional | 8 (2%) | 6 (6%) |  | 5 (2%) | 1 (2%) |  |
| Metastatic | 18 (6%) | 8 (8%) |  | 6 (3%) | 2 (4%) |  |

* STAS – Tumor Spread Through Air Spaces

**Table S2: Multivariable Predictors of Mortality**

| **Risk Factors** | **HR (95% CI)** | **p-value** |
| --- | --- | --- |
| STAS | 0.9 (0.4-2.2) | 0.807 |
| Interstitial Fibrosis | 3.5 (0.4-28.7) | 0.245 |
| Pathologic Stage |  |  |
| I | Ref |  |
| IIa | 5.7 (1.2-27.5) | 0.028 |
| IIb | 2.6 (1.1-6.2) | 0.026 |
| IIIa | 5.5 (1.1-27.8) | 0.040 |
| PD-L1 Expression |  |  |
| <1% | Ref |  |
| 1-50% | 0.4 (0.2-0.9) | 0.048 |
| >50% | 0.5 (0.1-1.6) | 0.250 |

* STAS – Tumor Spread Through Air Spaces; PD-L1 – Programmed Death Ligand 1

**Supplemental Figures**

**Figure S1: Histologic Analysis**

(A) Association between histology and presence of STAS, (B) Association between histologic subtype and presence of STAS


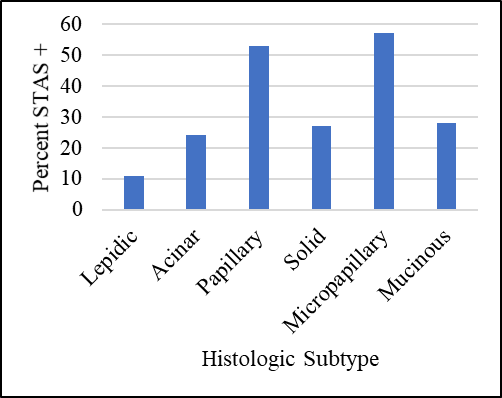

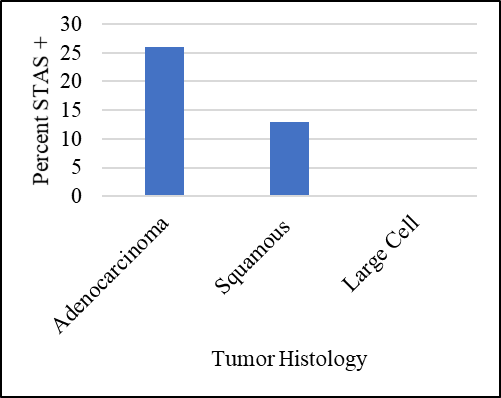


**Figure S2: Association between Tumor/Margin Ratio, STAS, and Recurrence**


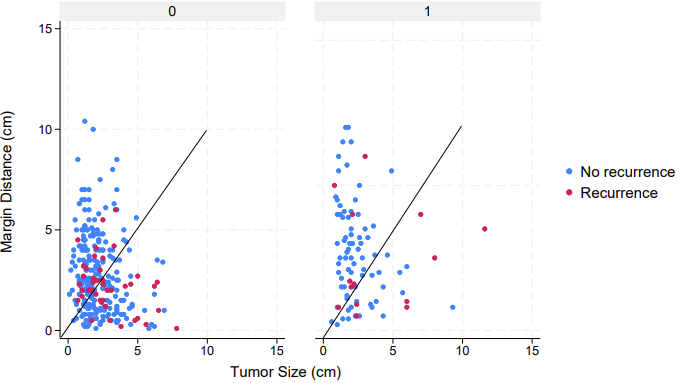


STAS +

No STAS

Tumor/margin ratio <1
